# Antimicrobial resistance and integron gene cassette arrays in commensal *Escherichia coli* from human and animal sources in IRI

**DOI:** 10.1186/s13099-016-0123-3

**Published:** 2016-08-30

**Authors:** Roohollah Kheiri, Leili Akhtari

**Affiliations:** 1 Molecular Microbiology, Quality Control Office, Alborz Province Water and Wastewater Company, Karaj, Alborz Islamic Republic of Iran; 2 Water Treatment Plant, Tehran Water and Wastewater Supply and Treatment Company, Tehran, Islamic Republic of Iran

**Keywords:** Antimicrobial resistance, *Escherichia coli*, Gene cassette, Integrons

## Abstract

**Background:**

The human and animal intestinal tract harbors a complex community of microbes which enables bacteria to inherit antibiotic resistance genes. The aims of this study were to investigate clonality, antimicrobial resistance, prevalence and gene cassette arrays of class I and II integrons among commensal *Escherichia coli* from human and animals.

**Methods:**

A total of 200 *E. coli* isolates from human, chicken, cattle, and sheep were isolated followed by phenotypic antibiotic susceptibility testing and detection of class I and II integrons gene cassettes arrays. The clonal relationship of the isolates were analyzed by (GTG)_5_-PCR.

**Results:**

Of 200 isolates, 136 isolates were multi drug resistance (MDR) including 47, 40, 31 and 18 isolates from chicken, human, cattle and sheep, respectively. Class I integron was detected in 50, 38, 6 and 16 %, while class II was detected in 26, 8, 0 and 4 % of chicken, human, cattle and sheep isolates, respectively. Variable regions were amplified and sequenced. Cassette arrays in class I integrons were: *dfrA1, dfrA5, dfrA7, dfrA12, aadA1, dfrA17 aadA1, aadA22, aadB*–*aadA2* and *dfrA12*–*orfF*–*aadA2*, and for class II, *dfrA1*-*sat*-*aadA1,* and *sat*-*sat1*-*aadA1* were detected. Six class I and three class II positive strains did not produce any amplicons for variable region. Integron-positive isolates showed higher rate of resistance to streptomycin and trimethoprim–sulphamethoxazole, especially in chicken isolates which were fed antibiotics. Low similarity and great genetic diversity of class I and II integrons carrying isolates indicated no clonal relation.

**Conclusions:**

Integrons encoding for antibiotic resistance are significantly present among non-pathogenic commensal *E. coli*, especially from the hosts medicated by antibiotics. Uncontrolled use of antibiotics will increase the numbers of multiple drug resistant isolates and integrons prevalence.

## Background

*Escherichia coli* is a major member of the human and animal gut’s normal microflora. Although commensal *E. coli* strains are non-pathogen, pathogenic types of *E. coli* including enterotoxigenic (ETEC), enterohemorrhagic (EHEC), enteroaggregative (EAEC), enteroinvasive (EIEC), and enteropathogenic (EPEC) can cause intestinal diseases [1].

Carrying resistance plasmids and the ability of transferring resistance, *E. coli* is a potential reservoir for antimicrobial resistance genes which plays an important role in the ecology of antimicrobial resistance of bacterial populations [2]. In addition, enteric fecal flora from food producing animals such as chicken, cattle and sheep may transfer antimicrobial resistance to human pathogens [3]. Exposure to resistant bacteria via the food-chain has gained increased attention because the presence of resistant bacteria in food and water might have an impact on the development and dissemination of antibiotic resistance among human bacterial pathogens [3]. Followed by receiving antibiotics, the emergence of *E. coli* isolates with multiple antibiotic-resistant phenotypes, involving resistance to various families of antibiotics, has been previously reported and it has been suggested that resistance in bacterial populations may spread from one ecosystem to another by lateral gene transfer, specifically integrons [4]. Integrons are bacterial genetic elements that allow the shuffling of smaller mobile elements called gene cassettes; they have been termed ‘a genetic construction kit for bacteria [5]. Integrons are involved in the evolution and spreading of antibiotic-resistance genes in enteric bacteria. The amino acid sequences of the integrases have been used as a basis for dividing integrons into ‘classes’, with those carrying *intI1* defined as ‘class I’, *intI2* as ‘class II’, *intI3* as ‘class III’, etc. Classes of integrons are widely found in gram-negative bacteria and are associated with the spread of antimicrobial drug resistance throughout the world. Therefore, isolates carrying integrons, can survive if exposed to antibiotics [6]. This mostly happens in clinical and veterinary isolates, which will lead to resistance dissemination and failure of treatments. Apart from being carried by clinical strains, studies have reported the occurrence of integrons and gene cassettes in the enteric faecal flora of humans and animals [4]. Since many gene cassette arrays of integrons contain antimicrobial resistance (AMR) factors, the horizontal transfer of integrons through plasmids and transposons has been found to play an important role in the dissemination of AMR genes and development of multi-resistance in enteric tract, which will be a great health concern [7].

In fact, the main aims of the current study were to determine the prevalence of class I and II integrons and to characterize integron-associated gene cassette arrays in commensal *E. coli* from human and animal sources in IRI. In addition to the main aims, we aimed to determine the clonality of isolates and to evaluate the applicability of (GTG)_5_-PCR for differentiating and source tracking of the *E. coli* isolates from different hosts.

## Methods

### Samples collection

In this cross-sectional study, in August 2015, the authors collected the faecal samples from four species: human participants from Kavosh Medical Laboratory (located in Alborz province), chicken, cattle and sheep. Chicken enteric specimens were obtained from private animal breeding farm Qadir in Karaj city (suburb of Alborz province with geographic coordinate of 35.8840059, 50.9716793), while sheep and cattle enteric samples were collected from private facility Raeesi located in Zavvareh city (suburb of Isfahan province with geographic coordinate of 33.449974, 52.490830). Since faecal samples were collected from living animal, no animal was sacrificed.

Before sampling, oral and written information about the study was given to the human participants and owners of breeding farm and facility and an informed consent was obtained. Furthermore, Demographic data about the antibiotics consumptions were recorded. Human participants, cattle and sheep did not take any antibiotic for at least 4 weeks but chicken were fed fosbac (antibiotic for the treatment of systemic and respiratory bacterial diseases caused), trimethoprim-sulphamethoxazole, and oxytetracycline (to increase weight gain and feed conversion ratio in chickens).

For animal sampling, following autoclaving swabs in the capped tubes, they were inserted into the cloaca and rectum of animals in such a manner as to insure the collection of faecal material. The swabs and adhering faecal material were then placed in the tube and quickly shipped to laboratory. However, as mentioned before, human faecal samples were obtained by the participants who referred to Kavosh Medical Laboratory.

Following sampling, a total of 200 faecal samples (50 samples from either of species) were collected and shipped to the central laboratory of Alborz province water and wastewater Company for isolation of *E. coli.*

### Bacterial isolates

To isolate *E. coli*, faecal samples were inoculated to lauryl sulphate tryptose (LST) broth (Merck KGaA) followed by *E. coli* (EC) broth (Merck KGaA) at 44.5 °C and streaked on eosin methylene blue agar (EMB) agar (Merck KGaA). Colonies showing metal sheen were considered as presumptive *E. coli* isolates and were subjected to IMV*i*C (Merck KGaA), glucuronidase and tryptophanase tests for final confirmation [8].

### Antimicrobial susceptibility testing

Phenotypical antibiotic susceptibility was tested applying Pad tan Teb (Tehran, Iran) disks by Kirby-Bauer disk diffusion method on Mueller–Hinton agar plates according to the guidelines of Clinical and Laboratory Standards Institute [9]. A panel of 24 antibiotic discs were used as follows: ceftazidime (30 μg), cefotaxime (30 μg), ceftriaxone (30 μg), ceftizoxime (30 μg), cephalothin (30 μg), cefazolin (30 μg), cephalexin (30 μg), trimethoprim/sulfamethoxazole (25 μg), amoxicillin/clavulanic (20/10 μg), piperacillin (100 μg), ampicillin. (10 μg), streptomycin (10 μg), tobramycin (100 μg), Amikacin (30 μg), kanamycin (30 μg), neomycin (30 μg), gentamycin. (10 μg), norfloxacin (10 μg), ciprofloxacin (5 μg), nalidixic acid (30 μg), levofloxacin (5 μg), chloramphenicol (10 μg), tetracycline (10 μg), and doxycycline (10 μg). Isolates resistant to at least one antimicrobial agent were tested for the presence of class I and II integrons genes by polymerase chain reaction (PCR).

### DNA extraction and PCR assay

To extract the genomic DNA, bacterial cells were centrifuged at 2500 revolutions per minute (rpm) for 15 min discarding the supernatant, following manufacturer’s protocol (Bioneer’s *AccuPrep*^®^ Genomic DNA Extraction Kit); DNA from all isolates was extracted.

To detect class I and II integrases and to develop a duplex PCR assay, two sets of primer were designed using Allele ID v7.6 software (PREMIER Biosoft, United States). The sequences and amplicons sizes of the primer sets are shown in Table 1. To validate the primer sets, two strains harbouring class I and II integrase genes were obtained from Baqiyatallah University of Medical Sciences‚ Molecular Biology Research Center‚ Tehran‚ Iran.

**Table 1:** Primer sets used for amplification of class I and II integrons

Gene target	Sequence (5′–3′)	References
*Int I. F*	TCTCGGGTAACATCAAGG	This study
*Int I. R*	GTTCTTCTACGGCAAGGT	This study
*Int II. F*	CACGGATATGCGACAAAAAGGT	This study
*Int II. R*	GTAGCAAACGAGTGACGAAATG	This study
Class I integron variable region	GGCATCCAAGCAAG	Levesque et al. [9]
Class I integron variable region	AAGCAGACTTGACCTGA	Levesque et al. [9]
Class II integron variable region	GATGCCATCGCAAGTACGAG	White et al. [10]
Class II integron variable region	CGGGATCCCGGACGGCATGCACGATTTGTA	White et al. [10]

The gene amplification protocol was performed by Applied Biosystems instruments (ABI) verity 96 well thermal cycler with the temperature profile as follows: initial denaturation (94 °C for 5 min), followed by 30 cycles of denaturation (94 °C for 20 s), annealing (30 s at 60 °C), and extension (72 °C for 1 min); and then a final extension (72 °C for 10 min). Isolates carrying class I and II integrase genes were further characterized through PCR for variable regions under the conditions described previously [10, 11]. Each cassette gene PCR product with a distinctive size was sequenced by Bioneer Sequencing Service. PCR products of the same size were restricted with FastDigest *Rsa*I (Thermo Scientific) and *Hin*fI (Thermo Scientific) enzymes. Two representative products of each distinct restriction fragment length polymorphism were purified by *AccuPrep*^®^ PCR Purification Kit (Bioneer, South Korea). Contiguous sequences were created from single sequence reads by using the MEGA 5 sequence assembly program and compared with GenBank^®^ (http://www.ncbi.nlm.nih.gov/genbank).

### Statistical analysis

The SPSS software (version 19) was used for statistical analysis. The association between the presence of *dfr* cassette array (in both class I and II) and trimethoprim/sulfamethoxazole (SXT) resistance, in addition to *aad* cassette array (in both class I and II) and streptomycin resistance were determined by χ^2^ or Fisher’s exact test. A *p* value of <0.05 was considered statistically significant.

### Rep-PCR fingerprinting

(GTG)_5_-PCR was applied for molecular fingerprinting of the isolates carrying variable regions of integrons under the conditions described before [12]. Gel images were normalized, and fingerprints were assigned to isolates, with Bio-Rad’s Image Lab™ 4.0 software. For cluster analysing, the data were converted to a binary matrix and using Mesquite (version 2.75) and PAUP (version 4.beta 10) softwares, similarity trees were generated using the unweighted-pair group method (UPGMA) using average linkages on the basis of 80 % similarity.

## Results

### Bacterial isolates

Following standard *E. coli* isolation procedure, 200 isolates (one isolate per faecal sample) were collected and included in the present study.

### Antimicrobial resistance and integrons cassette arrays

Antibiotic susceptibility results showed, 136 isolates were MDR (multidrug-resistant). As shown in Table 2, fourteen isolates were resistant to only one antibiotic category, while 136 isolates were resistant to more than two categories (MDR isolates). The number of MDR isolates from chicken, human, cattle, and sheep were 50, 50, 48 and 38, respectively. Of the 136 *E. coli* isolates, 55 isolates carried *IntI* and 19 isolates carried *IntII,* while nine isolates carried both classes. Class I integrase gene was detected in 50 % (25/50), 38 % (19/50), 6 % (3/50) and 16 % (8/50), while class II integrase gene was detected in 26 % (13/50), 8 % (4/50), 0 % (0/50) and 4 % (2/50) of chicken, human, cattle and sheep isolates, respectively. Among the isolates, 14 % (7/50) of chicken and 4 % (2/50) of sheep harboured both classes of integrase genes simultaneously.

**Table 2:** Sources and the number of resistant isolates to antimicrobial categories

No. of resistant categories	No. of chicken isolates	No. of human isolates	No. of cattle isolates	No. of sheep isolates	Sum
Resistant to 7 categories	0	0	0	0	0
Resistant to 6 categories	11	0	1	1	13
Resistant to 5 categories	17	6	0	0	23
Resistant to 4 categories	10	12	7	4	33
Resistant to 3 categories	9	22	23	13	67
Resistant to 2 categories	3	10	17	20	50
Resistant to 1 category	0	0	2	12	14
Resistant to 0 category	0	0	0	0	0
Resistant to 2 or more categories	47	40	31	18	136

Following sequencing and comparing with GenBank, nine and four different gene cassette arrays were found in classes I and II integrons, respectively (Tables 3, 4). However, in six class I integron positive and three class II integron positive isolates, the cassette arrays could not be detected by PCR.

**Table 3:** Gene cassette arrays of class I integrons in *E. coli* from different species

Species/cassette array	No. (%) of the human isolates carrying gene cassette array	No. (%) of the chicken isolates carrying gene cassette array	No. (%) of the sheep isolates carrying gene cassette array	No. (%) of the cattle isolates carrying gene cassette array	Total^a^
*dfrA1*	1 (2)	4 (8)	0 (0)	1 (2)	6
*dfrA5*	0 (0)	3 (6)	1 (2)	0 (0)	4
*dfrA7*	1 (2)	2 (4)	0 (0)	0 (0)	3
*dfrA12*	2 (4)	3 (6)	1 (2)	1 (2)	7
*aadA1*	3 (6)	10 (20)	4 (8)	1 (2)	18
*aadA4*	0 (0)	2 (4)	1 (2)	0 (0)	3
*dfrA17*-*aadA1*	0 (0)	2 (4)	0 (0)	0 (0)	2
*aadA1*-*aadB*	1 (2)	2 (4)	0 (0)	0 (0)	3
*dfrA12*–*orfF*–*aadA2*	0 (0)	2 (4)	1 (2)	0 (0)	3
Total	8 (16)	30 (60)	8 (16)	3 (6)	49

^a^The number of the isolates carrying *intI*, is 55, however, in six class I integron positive isolates, the cassette arrays could not be detected by PCR

**Table 4:** Gene cassette arrays of class II integrons in *E. coli* from different species

Species/cassette array	No. (%) of the human isolates carrying gene cassette array	No. (%) of the chicken isolates carrying gene cassette array	No. (%) of the sheep isolates carrying gene cassette array	No. (%) of the cattle isolates carrying gene cassette array	Total^a^
*dfrA1*	1 (2)	1 (2)	2 (4)	0 (0)	4
*dfrA1*-*sat2*-*aadA1*	0 (0)	2 (4)	1 (2)	1 (2)	4
*dfrA1*-*sat1*-*aadA1*	1 (2)	3 (0)	0 (0)	0 (0)	4
*at*-*sat1*-*aadA1*	1 (2)	2 (4)	0 (0)	1 (2)	4
Total	3 (6)	8 (16)	3 (6)	2 (4)	16

^a^The number of the isolates carrying *intII*, is 19, however, in three class II integron positive isolates, the cassette arrays could not be detected by PCR

Based on statistical analysing, it could be inferred that there was a significant association between *dfr* cassette arrays and SXT resistance (*p* < 0.001) as well as the resistance against the streptomycin (10 μg) which may be conferred by *aad* cassette arrays (*p* < 0.002).

### DNA fingerprinting analysis

**Fig. 1:**
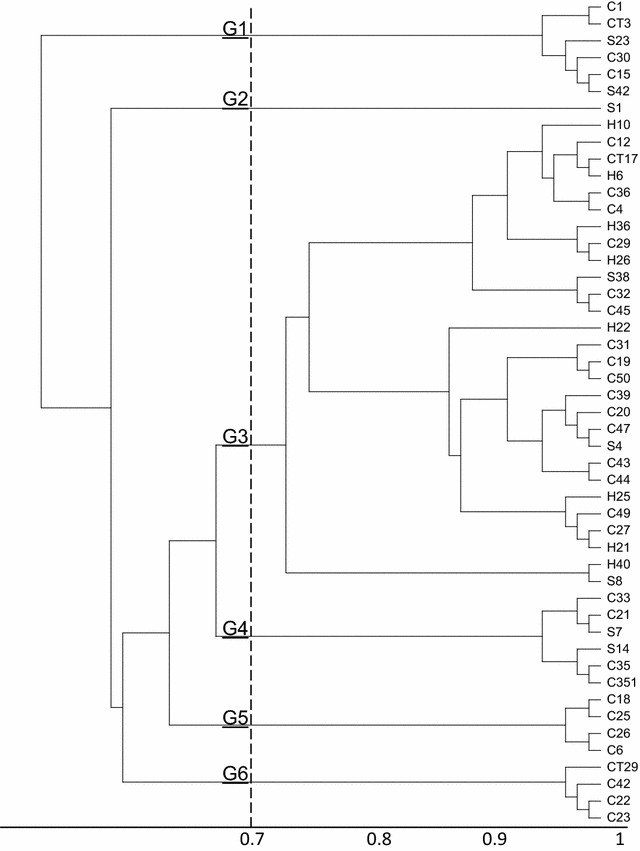
Dendrogram analysis of DNA fingerprinting obtained from 49 *E. coli* strains carrying gene cassette arrays of class I integron by (GTG)_5_ polymerase chain reaction. The *letters* and *digits* shown *right side* of the dendrogram indicate the code of isolates. *H* isolate from human; *C* isolate from chicken; *S* isolate from sheep; *CT* isolate from cattle

**Fig. 2:**
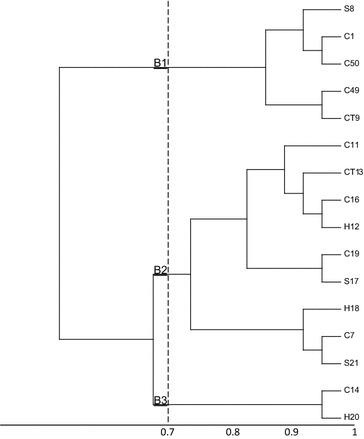
Dendrogram analysis of DNA fingerprinting obtained from 14 *E. coli* strains carrying gene cassette arrays of class II integron by (GTG)-PCR. The *letters* and *digits* shown *right side* of the dendrogram indicate the code of isolates. *H* isolate from human; *C* isolate from chicken; *S* isolate from sheep; *CT* isolate from cattle

(GTG)_5_ Fingerprints of commensal *E. coli* isolates carrying gene cassette arrays of class I and class II integrons was determined using the (GTG)_5_-PCR assay. (GTG)_5_-PCR of 49 isolates carrying gene cassette arrays of class I integron, yielded complex banding patterns consisting four to twelve bands ranging in size from 555 base pair (bp) to 3300 bp. However, (GTG)_5_-PCR of 14 isolates carrying gene cassette arrays of class II integrons, yielded five to twelve bands ranging in size from 530 to 3200 bp. To evaluate the clonal relation, dendrograms were constructed from (GTG)_5_ profiles of the isolates (Figs. 1, 2). At a cut off of 80 % similarity, UPGMA clustering separated all the isolates into various clusters. The 49 isolates carrying class I integron were assigned into six major clusters (G1–G6) and the 14 isolates carrying class II integron were assigned into three major clusters (B1–B3).

Based on the results of the cluster analysis, it was observed that isolates with the same gene cassette arrays were not assigned to a given cluster and in some cases the isolates with different cassette arrays were assigned to the same cluster; therefore, we came to this conclusion that no cassette array could be attributed to a specific (GTG)_5_ cluster.

In addition to the main aims, we analyzed the applicability of the (GTG)_5_-PCR for differentiating of the host animal. As it is observed, *E. coli* from different sources are assigned in different clusters confirming the low discriminatory power and unsuitability of rep-PCR methods for microbial source tracking.

## Discussion

Antimicrobial resistance in clinical isolates has become a major public health concern [13]. Most studies are directed to clinical isolates because such isolates have probably been subjected to a considerably higher antibiotic selection pressure or to broader spectrum antimicrobials [3]. However, few studies have subjected the AMR and integron prevalence of non-pathogenic commensal bacteria. Integrons are known to be primary source of transferable resistance genes and are suspected to serve as reservoirs of antimicrobial resistance genes within microbial populations [14, 15]. The present study characterized class I and II integrons and their gene cassettes arrays conferring resistance to several categories of antibiotics in commensal *E. coli* isolates from different species. Of the 200 *E. coli* isolates from different species, 136 isolates were MDR, 55 isolates carried class I, 19 isolates carried class II, and 9 isolates carried both of the classes. The distribution of gene cassette arrays among human, sheep, and cattle isolates was similar (Tables 3, 4), except from the chicken isolates. Our demographic data showed among the species, just chicken were fed fosbac, trimethoprim-sulphamethoxazole, and oxytetracycline, both for treatment and as growth factors. Therefore, it is not surprising to observe higher integrons prevalence (50 % for class I, 26 % for class II, and 14 % for both classes) and more MDR isolates in *E. coli* from chicken (100 % MDR).

Many gene cassette arrays able to confer resistance to several antimicrobial classes have been described in class I and II integrons, but those more frequently found are *dfrA*, *aadA,* and, for class II integrons, *sat* [16, 17]. Our PCR results showed, *dfr* and *aad* were the most prevalent genes cassette arrays which were in agreement with Daikos et al. [18]. Aminoglycoside adenylyl transferases encoded by *aad* family cassette arrays causes streptomycin/spectinomycin resistance, considering the Table 5, we found a significant association between isolates carrying family of *aad* cassette arrays and phenotypically resistance against streptomycin. The other common resistance was observed against trimethoprim/sulfamethoxazole (SXT), which could be explained by *drf* gene cassette arrays, because these arrays encode for dihydrofolate reductase enzymes that can yield to trimethoprim resistance. Considering the demographic data of drug consumption as well as drug resistance, the hypothesis of antibiotic mediated resistance could be deduced, because the chicken was the only species which received SXT and it is observed that the *dfr* cassette arrays (*dfrA1, dfrA5, dfrA7, dfrA12, dfrA17, dfrA12*–*orfF*–*aadA2,* and *dfrA1*-*sat*-*aadA1*) and consequently resistance to SXT, were much more prevalent in this species [19].

**Table 5:** Integrons gene cassette arrays and phenotypically resistant isolates

Integrons and cassette/arrays	No. of the isolates carrying gene cassette array	No. of isolates phenotypically resistant to antibiotics									
		SXT (25 μg)	CAZ (30 μg)	S (10 μg)	GM (10 μg)	AN (30 μg)	K (30 μg)	AMP (10 μg)	PRL (100 μg)	NA (30 μg)	C (30 μg)
Class I integrons	49	25	5	33	7	0	3	23	4	9	5
*dfrA1*	6	4	1	2	0	0	0	4	0	1	1
*dfrA5*	4	2	0	1	0	0	0	3	0	1	0
*dfrA7*	3	2	1	2	0	0	0	2	0	0	0
*dfrA12*	7	3	0	4	0	0	0	4	1	2	0
*aadA1*	18	7	2	15	3	0	0	3	2	3	1
*aadA4*	3	2	0	2	2	0	0	3	0	1	0
*dfrA17*-*aadA1*	2	2	0	2	1	0	2	1	0	0	2
*aadA1*-*aadB*	3	1	1	2	1	0	1	2	1	0	1
*dfrA12*–*orfF*–*aadA2*	3	2	0	3	0	0	0	1	0	1	0
Class II integrons	16	9	7	10	5	0	3	6	3	4	2
*dfrA1*	4	3	1	1	0	0	0	2	1	1	0
*dfrA1*-*sat2*-*aadA1*	4	2	2	3	2	0	1	1	0	0	1
*dfrA1*-*sat1*-*aadA1*	4	1	1	2	1	0	2	3	2	2	1
*at*-*sat1*-*aadA1*	4	3	3	4	2	0	0	0	0	1	0
Total	65	34	12	43	12	0	6	29	7	13	7

*SXT* trimethoprim/sulfamethoxazole, *CAZ* ceftazidime, *S* streptomycin, *GM* gentamycin, *AN* amikacin, *K* kanamycin, *AMP* ampicillin, *PRL* piperacillin, *NA* nalidixic acid, *C* chloramphenicol

As mentioned before, the phenotypic resistance to a specific antibiotic was observed in most of the isolates carrying the corresponding gene cassette which was in agreement with Hall et al. [19]; Barlow et al. [20]; Kor et al. [21], and Li et al. [22]. However, it was also evident that some integron-carrying organisms had reduced susceptibility not only to antimicrobial agents for which the respective gene cassettes were contained in but also to other classes of agents for which no or very little number of genes were contained within the integrons [23].

Except from trimethoprim/sulfamethoxazole and streptomycin, the other proportionally high resistance was against ampicillin, but as listed in the Table 4, ampicillin resistance was completely distributed among the isolates carrying cassette arrays, indicating the ineffectiveness of the integrons for ampicillin resistance. Apparently resistance mechanisms or a considerable number of antibiotic resistance genes were located outside the integrons either on chromosomes or plasmids [6], therefore, not all the resistance profile of the isolates could be explained by the expression of the gene cassettes found within the integrons [24]. Nevertheless, some isolates carrying integrons were susceptible to the corresponding antibiotic. This finding may be explained by the lack of expression of gene cassettes contained in integrons, as described by Kim et al. [24].

Based on our results, resistance against ceftazidime, gentamycin, amikacin, kanamycin, piperacillin, nalidixic acid and chloramphenicol were not significance and cannot be attributed to the integrons. However, Moghaddam et al. identified all of *E. coli* strains isolated from human urine to be multi-drug resistant and PCR analysis revealed that 97 % of strains carried *IntI1* gene [25]. In another study, Tajbakhsh et al. reported that all *E. coli* strains isolated from different fish fields of Chaharmahal Va Bakhtiari, Iran, were multidrug resistant and 100 % resistance against the ciprofloxacin, chloramphenicol, gentamycin, ampicillin and tetracycline was observed [26]. Since fish field aquacultures, as an unselective media, can receive all kinds of contaminations, the antimicrobial resistance of their report can be generalized to the public health of the community, confirming the severity of antimicrobial resistance among commensal bacteria in Iran which can be explained by high consumption of antibiotics [26]. It is noteworthy to mention that based on available data, consumption of antibiotics in livestock and poultry in Iran is higher than developed countries [27].

Our results confirmed Kang’s et al. survey in which commensal *E. coli* isolated strains from enrofloxacin and norfloxacin medicated poultry were compared to *E. coli* from swine, which were not fed by the mentioned above agents, whereby they found the poultry-originated *E. coli* to be much more resistant than the swine ones because of higher prevalence of *dfrA12*-*orfF*-*aadA2* gene cassette array [28]. These results warn the increasing rate of antibiotic resistance because of antibiotic therapy.

As a result of universal intensive chicken antibiotic feeding, the rate of AMR and integrons prevalence were to some extent similar worldwide. Cavicchio et al. isolated 299 *E. coli* from avian source and found class I and II, in 49.8 and 10.4 % of the samples while thirteen strains (4.3 %) were positive for both classes [23]. Our molecular results are also comparable to Cavicchio et al. because as they reported, the most common gene cassette arrays identified in class I integron-positive isolates belonged to the *aadA* (90/149 isolates, 60.4 %) and the *dfrA* (50/149 isolates, 33.6 %) gene families, with 5 and 4 different variants, respectively. However, the prevalence of *aadA* and *dfrA* cassette arrays in class II integrons are incompatible to our results, because Cavicchio et al. identified *aadA* and *dfrA* in 67.7 and 64.5 % of the class II integron positive isolates [23].

Elizabeth et al. studied *E. coli* isolates from chicken litter and found class I integron genes in 52.63 % of the isolates which is compatible to our results [29]. Furthermore, they found *dfrA1* and *aadA1* as the most prevalent gene cassette arrays.

Vasilakopouloue et al. [30] evaluated the prevalence of class I integron and gene cassettes arrays in *E. coli* of poultry and human and found the integron carriage rate for poultry isolates was 49.2 %, for hospital isolates was 26.2 % and for healthy people was 11.1 %. While *aad* and *dfr* families were the most prevalent cassette arrays. Vasilakopouloue’s survey confirmed our results (poultry integron rate) and showed a difference between AMR of hospitalized and healthy people which can be attributed to drug consumption by hospitalized patients.

Karczmarczyk et al. studied molecular characterization of 100 multidrug-resistant *E. coli* isolates from Irish Cattle Farms [31]. In their study, Karczmarczyk et al. found twenty-seven (27 %) isolates harboured class I integrase (which is higher than class I integron rate in *E. coli* from cattle included in our study), while the variable regions of these integrons contained *aadA12, aadB*-*aadA1, blaOXA*-*30*-*aadA1, dfrA1*-*aadA1, and dfrA7*. Class II integrons were identified less frequently (4 %) and contained the gene cassette array *dfrA1*-*sat1*-*aadA1*. Moreover, Karczmarczyk et al. found high resistance of streptomycin (100 %), tetracycline (99 %), ampicillin (82 %), and sulfonamide (98 %) which was approximately similar to the results reported by Pourtaghi et al. in *E.coli* isolated from calves in Iran, except from the sulfonamide. Moreover, Pourtaghi et al. reported the resistance of 91.8, 93, 100 and 73.2 % for streptomycin, tetracycline, penicillin, and sulfonamide respectively [32].

Of course the prevalence of class I integrons investigated by Oosterik et al. is half of our report since they found *intI* gene in 21.6 % of *E. coli* isolated from poultry faeces; however, in their report there is no demographic record of antibiotic prescription to explain such difference [33].

Following amplifying the variable region of the integrons, six integron class I positive isolates and three integron class II positive isolates produced no amplicons when PCR was repeatedly preformed for amplification of gene cassette arrays. Sunde had the same experience [3]. She could not amplify variable region of class I integrons in 10 *intI* positive strains as well as Malek et al. who did not manage to amplify the cassette regions of three class I integrons isolates [6]. However, Rearrangements, lack of 3′-conserved segment(s), absence of primer hybridization site(s), or presence of early stop codon may explain such phenomenon [34].

The antibiotic therapy of *E. coli* infections is now threatened by the emergence of antimicrobial resistance. The dissemination of resistance is associated with genetic mobile elements, such as plasmids, that may also carry virulence determinants [35]. Despite the difference in the evolution of antimicrobial resistance and virulence factors, they share some common characteristics [35]. From a biological point of view, both processes are necessary for bacteria to survive under adverse conditions. Virulence mechanisms are necessary to overcome host defence systems, and the development of antimicrobial resistance is essential to enable pathogenic bacteria to overcome antimicrobial therapies and to adapt to and survive in competitive and demanding environments [36]. An example of involvement of antimicrobial resistance and virulence can be observed in *E. coli* ST131 (O25:H4). Producing CTX-M-15 extended-spectrum β-lactamase (ESBL), this strain has emerged internationally as a multidrug-resistant (MDR) pathogen. Thus, *E. coli* isolates able of producing CTX-M-type ESBL and *E. coli* ST131 have been found to exhibit a wide array of virulence factors [36]. In a study conducted by Asadi et al. in Iran, statistically significant association between *fimH* gene and resistance to ciprofloxacin, nalidixic acid, and cotrimoxazole was found as well as the associations between *ibeA* gene and amikacin, elucidating the role of antimicrobial resistance both on attenuation of antibiotic therapy and augmentation of the pathogenesis [37].

In addition to the study of antibiotic resistance and integrons, we analyzed the genetic diversity and the applicability of the (GTG)_5_-PCR for differentiating of the host animals, while we found it unsuitable for tracking, as the result of high degree of diversity and low intra-species similarity among the *E. coli* isolates. Furthermore, dendrogram generated by (GTG)_5-_ profiles showed that isolates from different species were distributed in different clusters, confirming inapplicability of (GTG)_5_-PCR as reliable tracking tool. Although, Dombeck et al., reported BOX-PCR and REP-PCR methods are reliable tools to separate the human *E. coli* isolates from the nonhuman isolates [38] and Mohapatra et al., noted the acceptable average rate of correct classification (ARCC) of (GTG)_5_-PCR for *E. coli* source tracking [39], however, it should be considered that controversial reproducibility of PCR, differences in DNA concentration and thermal condition may lead to produce fake bands or remove weak bands, therefore, questioning the applicability of rep-based PCR for source tracking. For more efficient finger printing and subtyping, it is recommended to apply pulse field gel electrophoresis (PFGE) which is a reliable and highly discriminating method and has been considered to be the “gold standard” of typing methods. Through the establishment of PulseNet [40], use of PFGE has had a major impact on pathogen subtyping and outbreak investigation [41].

## Conclusion

Our findings highlighted the importance of the commensal microflora of animals as a reservoir of transferable genes. We concluded that integrons carrying gene cassettes encoding for antibiotic resistance are significantly present among non-pathogenic commensal *E. coli*, especially from the hosts medicated by antibiotics. Uncontrolled use of antibiotics would increase the numbers of MDR isolates and integrons prevalence, which after a while, it could be a significant public health concern.

## Abbreviations

*Dhfr*: dihydrofolate reductase
aad: aminoglycoside adenylyltransferase determinant
*E. coli*: *Escherichia coli*
ETEC: enterotoxigenic *E. coli*
EHEC: enterohemorrhagic *E. coli*
EAEC: enteroaggregative *E. coli*
EIEC: enteroinvasive *E. coli*
EPEC: enteropathogenic *E. coli*
MDR: multiple drug resistant
AMR: antimicrobial resistance
IRB: Institutional Review Board
IACUC: Institutional Animal Care and Use Committee
LST: lauryl tryptose
EMB: eosin methylene blue agar
BLAST: blast basic local alignment search tool
PCR: polymerase chain reaction
ABI: applied biosystems instruments
SXT: trimethoprim/sulfamethoxazole
PAUP: phylogenetic analysis using parsimony
UPGMA: unweighted-pair group method
CAZ: ceftazidime
S: streptomycin
GM: gentamycin
AN: amikacin
K: kanamycin
AMP: ampicillin
PRL: piperacillin
NA: nalidixic acid
C: chloramphenicol
